# Chronic Low-Calorie Sweetener Use and Risk of Abdominal Obesity among Older Adults: A Cohort Study

**DOI:** 10.1371/journal.pone.0167241

**Published:** 2016-11-23

**Authors:** Chee W. Chia, Michelle Shardell, Toshiko Tanaka, David D. Liu, Kristofer S. Gravenstein, Eleanor M. Simonsick, Josephine M. Egan, Luigi Ferrucci

**Affiliations:** Intramural Research Program, National Institute on Aging, National Institutes of Health, Baltimore, Maryland, United States of America; McMaster University, CANADA

## Abstract

**Introduction:**

Low-calorie sweetener use for weight control has come under increasing scrutiny as obesity, especially abdominal obesity, remain entrenched despite substantial low-calorie sweetener use. We evaluated whether chronic low-calorie sweetener use is a risk factor for abdominal obesity.

**Participants and Methods:**

We used 8268 anthropometric measurements and 3096 food diary records with detailed information on low-calorie sweetener consumption in all food products, from 1454 participants (741 men, 713 women) in the Baltimore Longitudinal Study of Aging collected from 1984 to 2012 with median follow-up of 10 years (range: 0–28 years). At baseline, 785 were low-calorie sweetener non-users (51.7% men) and 669 participants were low-calorie sweetener users (50.1% men). Time-varying low-calorie sweetener use was operationalized as the proportion of visits since baseline at which low-calorie sweetener use was reported. We used marginal structural models to determine the association between baseline and time-varying low-calorie sweetener use with longitudinal outcomes—body mass index, waist circumference, obesity and abdominal obesity—with outcome status assessed at the visit following low-calorie sweetener ascertainment to minimize the potential for reverse causality. All models were adjusted for year of visit, age, sex, age by sex interaction, race, current smoking status, dietary intake (caffeine, fructose, protein, carbohydrate, and fat), physical activity, diabetes status, and Dietary Approaches to Stop Hypertension score as confounders.

**Results:**

With median follow-up of 10 years, low-calorie sweetener users had 0.80 kg/m^2^ higher body mass index (95% confidence interval [CI], 0.17–1.44), 2.6 cm larger waist circumference (95% CI, 0.71–4.39), 36.7% higher prevalence (prevalence ratio = 1.37; 95% CI, 1.10–1.69) and 53% higher incidence (hazard ratio = 1.53; 95% CI 1.10–2.12) of abdominal obesity than low-calorie sweetener non-users.

**Conclusions:**

Low-calorie sweetener use is independently associated with heavier relative weight, a larger waist, and a higher prevalence and incidence of abdominal obesity suggesting that low-calorie sweetener use may not be an effective means of weight control.

## Introduction

The worldwide onset of obesity over the previous 3 decades shows no sign of slowing with one billion adults projected to have obesity by 2025 [1]. In the United States, the dramatic increase in obesity measured by body mass index (BMI) has recently plateaued at 35% for men but continued to increase to 40% for women [2]. Similar trend was noted in the 60 years and older group [2]. The prevalence of abdominal obesity measured by waist circumference (WC) continues to rise in the US for both men and women, however [3]. Obesity and abdominal obesity in particular, are well-established risk factors for a host of medical conditions including diabetes, hypertension, and cardiovascular disease [4, 5]. Low-calorie sweetener use has long been recommended as a dietary strategy to reduce carbohydrate and total energy intake for weight and diabetes management [6, 7]. However, despite substantial increased low-calorie sweetener consumption after their introduction in the market, often in the form of diet soda, obesity remains entrenched [8, 9].

Consequently, the effectiveness of using low-calorie sweeteners for weight reduction and control has faced increasing scrutiny and remained controversial. Over the years, results from randomized controlled trials of low-calorie sweeteners for weight loss have been inconsistent [6, 10–18]. Findings from some observational studies suggest diet soda consumption may even increase risk of obesity, metabolic syndrome, and diabetes [19–26]. Recent studies suggested that these risks may be modified by dietary quality [27–29]. Nevertheless, findings have not been entirely uniform due to differences in how low-calorie sweetener consumption was assessed or in analytical models used. To clarify and expand on the potential consequences of low-calorie sweetener use, we evaluated up to 28 years of longitudinal data on diet and body composition collected in participants in the Baltimore Longitudinal Study of Aging (BLSA).

## Materials and Methods

### Study Population

The BLSA is an ongoing observational continuous-enrollment cohort study of normative aging in adults established in 1958 and currently conducted by the National Institute on Aging [30]. The BLSA participants are adult volunteers aged 20 years and older, recruited from the community and healthy at study entry. They undergo medical, physiological, and psychological examinations at age-specific visit intervals and return for follow-up visits for life. All participants provide written informed consent at each visit. The BLSA protocol has been approved by the Intramural Research Program of the US National Institute on Aging and the Institutional Review Board of the National Institute of Environmental Health Sciences.

The study sample includes 1454 men and women with at least one BLSA visit that included anthropometric measures and complete dietary record data beginning in 1984 when dietary records were first collected in both sexes. For each participant, the first visit, not necessary their visit at study entry, which included complete dietary record data and anthropometric measures was defined as the baseline visit. These 1454 participants contributed a total of 8268 person-visits of follow-up through 2012, with median follow-up of 10 years (range: 0–28 years).

### Measures

#### Anthropometric Measures

Height was measured in centimeters to the nearest one millimeter using a stadiometer; weight was measured in kilograms to the nearest 10^th^ of a kilogram; WC was measured in centimeters to the nearest one millimeter just below the rib cage where the waist tapers. BMI was defined as weight in kilograms divided by height in meters squared. Two different measures of adiposity were used: obesity defined as BMI ≥ 30 kg/m^2^; and abdominal obesity, an indicator of visceral fat deposition, defined as WC > 102cm for men and > 88cm for women [5, 31].

#### Assessment of Low-Calorie Sweetener Use

Dietary intake was assessed using a 7-day dietary record. The details of dietary collection and analyses have been described previously [32]. Diet data were collected at some visits from 1984 to 2008. Participants did not complete a dietary record at every visit to reduce participant burden, and all available dietary records were used. Low-calorie sweetener consumption was noted when consumption of food or drink containing low-calorie sweetener (aspartame, saccharin, acesulfame potassium, or sucralose) was recorded in the dietary record. This collection method identified low-calorie sweeteners found in all food products, not just diet soda.

#### Covariates

Covariates encompass socio-demographic factors including age, sex, age by sex interaction and race; behavioral factors known to impact weight such as physical activity (sedentary, low, moderate or high based on reported frequency and duration of vigorous and moderate physical activity) and smoking status (current smoker versus former and never smoker); dietary intake of specific nutrients (caffeine, fructose, protein, carbohydrate, and fat); and diet quality using the Dietary Approaches to Stop Hypertension (DASH) score. DASH score derived from consumption of total fat, saturated fat, protein, fiber, cholesterol, calcium, magnesium and potassium and which ranges from 0 to 9 with higher values signifying higher quality diet [33]. Year of visit and diabetes status determined from an oral glucose tolerance test (OGTT) and categorized as no, yes and pre-diabetes were accounted for in the analyses.

### Statistical Analysis

Associations between baseline low-calorie sweetener use and participant characteristics were assessed using chi-square tests for categorical variables and t-tests or Wilcoxon rank-sum tests for continuous variables. Associations between baseline low-calorie sweetener use and characteristics measured over all person-visits were assessed using unadjusted generalized estimating equations with robust standard errors [34].

#### Marginal Structural Models

**Fig 1:**
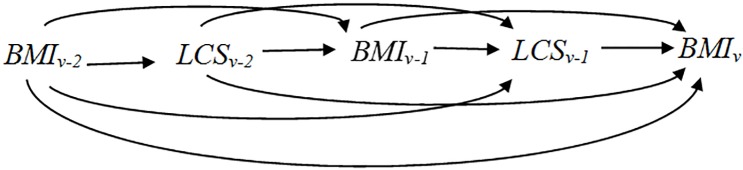
Schema for Marginal Structural Models. Adjusting for BMI at visit v-1 by including this term in the regression model would lead to bias because it is potentially in the causal pathway from low-calorie sweetener (LCS) use at visit v-2 to the study outcome (BMI at visit v here). However, failing to adjust for BMI at visit v-1 would lead to bias because it confounds the relation of LCS at visit v-1 with BMI at visit v. Marginal structural models overcome this problem by using inverse probability weights to adjust for confounders.

We used marginal structural models to determine the associations of low-calorie sweetener use history with study endpoints while accounting for time-independent and time-varying covariates. Similarly, we used marginal structural Cox proportional hazards models to determine the association of low-calorie sweetener use history with onset of obesity-related outcomes. Unlike conventional longitudinal analysis and Cox proportional hazards models, marginal structural models and marginal structural Cox proportional hazards models adjust for time-varying confounding using inverse probability of exposure weights rather than by including confounders in the regression model. The rationale for using marginal structural models and marginal structural Cox proportional hazards models in the current project is to appropriately account for time-varying confounding of an exposure history. Specifically, at each visit v for each participant, we operationalized low-calorie sweetener use history as the proportion of past visits (0 through v-1) in which the participant reported using low-calorie sweeteners. Thus, it is important to control for BMI (and other factors) at visits 0 through v-1 that may influence both the choice to use low-calorie sweeteners over time and the study outcome at visit v. However, if we were to include past BMI as a covariate as one would do in a conventional regression model, we would obtain biased results because we would be adjusting for a factor that may be in the causal pathway; but if we did not adjust for BMI, we would obtain biased results confounded by past BMI. The inverse probability weights allow us to overcome this catch-22 by adjusting for past BMI (and other factors) via standardization (i.e., the weights) rather than conditioning in a regression model. Marginal structural models and marginal structural Cox proportional hazards models can also be used to compute standardized associations of a baseline-measured exposure with study outcomes, as described in detail in Fig 1.

#### Baseline Low-calorie Sweetener Use

We used marginal structural models to determine the association between baseline low-calorie sweetener use and longitudinal outcomes [35]. Model 1 included visits after baseline as outcome visits and adjusted for baseline values of outcomes as confounders to minimize the potential of reverse causality. Additionally, Model 1 was adjusted for covariates. Inverse-probability weighting was used to account for confounders, study drop-out, and truncation by death [35, 36]. Specifically, inverse probability weights for low-calorie sweetener use, to adjust for confounders, were computed as the inverse of probabilities estimated using a logistic regression of low-calorie sweetener use on covariates. Inverse-probability weights for study drop-out were computed as the inverse of products of probabilities estimated using pooled logistic regression that included time-varying confounding factors, visit number and time between visits. This model included participants whose last study visit occurred before 2012 and who were not confirmed deceased by 2012. The weight used in analysis was the reciprocal of 1-probability of drop-out. Lastly, inverse-probability weights for death were computed as the inverse of products of probabilities of survival estimated using a Cox proportional hazards model that included time-varying confounding factors, visit number and time between visits. The weight used in analysis was the reciprocal of 1-probability of death. Intuitively, the product of 1-probability of death and 1-probability of drop-out can be interpreted as the probability of being alive and participating in the study. This weight helps to overcome selection bias due to death and drop-out. As a sensitivity analysis, we also included time (years from baseline)-by-low-calorie sweetener interactions to determine whether differences emerge early or later.

Cumulative incidence of obesity and abdominal obesity were also estimated and compared by baseline low-calorie sweetener use while accounting for the competing risk of death [37, 38]. Inverse-probability weights for baseline low-calorie sweetener use, study drop-out, and mortality were also used in a marginal structural Cox proportional hazards model to assess the associations of baseline low-calorie sweetener use with incident obesity and abdominal obesity [39].

#### Time-Varying Low-calorie Sweetener Use

Low-calorie sweetener use by participants fluctuated over time; therefore, we used marginal structural models to assess the relation of time-varying low-calorie sweetener use with longitudinal outcomes [35]. In Model 2, time-varying low-calorie sweetener use was operationalized as the proportion of visits since baseline at which low-calorie sweetener was used to summarize low-calorie sweetener use history. Inverse-probability weights for drop-out and mortality were computed as previously described; however, the covariates were also time-varying and included outcome measures assessed at previous visits [35, 36]. Inverse-probability weights for time-varying low-calorie sweetener use were computed using pooled logistic regression of low-calorie sweetener on year of visit, visit number, age, sex, age-by-sex interaction, race, current smoking status, values of time-varying dietary intake (caffeine, fructose, protein, carbohydrate, and fat) assessed at the same visit as low-calorie sweetener use, physical activity, diabetes status (no, yes, pre-diabetics), DASH score, and lagged (previous visit) low-calorie sweetener use, lagged outcomes, lagged diabetes status, and 2- and 3-way interactions between lagged low-calorie sweetener use, lagged diabetes status, sex, and time since previous visit as confounders. In other words, an outcome assessed at visit v was regressed on low-calorie sweetener history up to the visit prior to outcome assessment to rule out reverse causality. In Model 2, outcomes at visit v were regressed on low-calorie sweetener use history through visit v-1, hence minimizing reverse causality. Model 2 did not include outcomes assessed at baseline and adjusted for covariates. We additionally fit a marginal structural Cox proportional hazards model relating time-varying low-calorie sweetener use to incidence of obesity and abdominal obesity using the weights from Model 2. SAS version 9.2 and R version 3.1.1 were used for all analyses. P-value <0.05 was considered statistically significant for all analyses.

## Results

### Baseline Characteristics

At baseline, low-calorie sweetener users compared to non-users were on average 2 years younger, 59.5 ±15.8 years versus 61.8 ±15.8 years, respectively. Low-calorie sweetener users also had higher BMI, 26.4 ± 4.5 kg/m^2^ compared to 25.3 ± 4.0 kg/m^2^, as well as higher WC, 88.1 ±13.2 cm compared to 86.2 ± 12.0 cm. In addition, the low-calorie sweetener users had higher prevalence of obesity, abdominal obesity and type 2 diabetes (Table 1). Low-calorie sweetener use was not associated with differential total caloric intake, dietary macronutrient composition, physical activity level or smoking, but users consumed lower amounts of fructose and higher amounts of caffeine than non-users, but had similar diet quality. Median number of visits did not differ, but low-calorie sweetener users had a slightly longer follow-up than non-users (10.4 vs. 8.8 years).

**Table 1:** Comparison of baseline and longitudinal characteristics of low-calorie sweetener users versus non-users categorized by baseline usage.

	Baseline Characteristics			Characteristics over 28-year study period		
Characteristic	Low-calorie sweetener non-users (N = 785)	Low-calorie sweetener users (N = 669)	*P* Value	Low-calorie sweetener non-users (person-visits = 4324)	Low-calorie sweetener users (person-visits = 3944)	*P* Value
Age (year)	61.8 (15.8)	59.5 (15.8)	0.005	69.4 (15.1)	67.35 (15.5)	0.01
Male Sex	406 (51.7%)	335 (50.1%)	0.53	2295 (53.1%)	1969 (49.9%)	0.32
Race			0.14			0.64
• Caucasian	570 (72.6%)	516 (77.1%)		3435 (79.4%)	3217 (81.6%)	
• African-American	135 (17.2%)	96 (14.3%)		473 (10.9%)	402 (10.2%)	
• Other Race	80 (10.2%)	57 (8.5%)		416 (9.6%)	325 (8.2%)	
Body Mass Index (BMI) (kg/m^2^)	25.3 (4.0)	26.4 (4.5)	<0.001	25.5 (4.1)	26.8 (4.5)	<0.001
Waist Circumference (WC) (cm)	86.2 (12.0)	88.1 (13.2)	0.004	87.5 (12.2)	90.7 (13.2)	<0.001
Obesity (BMI ≥ 30 kg/m^2^)	93 (11.8%)	122 (18.3%)	0.001	540 (12.5%)	845 (21.4%)	<0.001
Abdominal Obesity (WC>102cm men; >88cm women)	126 (16.8%)	164 (25.2%)	<0.001	785 (19.5%)	1216 (32.8%)	<0.001
Energy Consumption (kcal/day)	1929.0 (539.2)	1924.2 (524.3)	0.86	1955.5 (581.8)	1929.7 (538.0)	0.38
Carbohydrate Consumption (g/day)	237.5 (75.4)	232.4 (77.5)	0.20	245.8 (83.4)	236.9 (80.6)	0.04
Protein Consumption (g/day)	76.9 (23.5)	78.9 (24.0)	0.10	77.3 (24.1)	79.2 (23.5)	0.10
Fat Consumption (g/day)	72.9 (26.5)	74.4 (26.7)	0.29	72.3 (27.7)	72.9 (27.3)	0.69
Caffeine Consumption (mg/day)	143.2 (145.2)	173.2 (239.0)	0.005	151.4 (203.0)	150.6 (188.0)	0.93
Fructose Consumption (g/day)	24.2 (13.2)	21.9 (11.5)	<0.001	25.1 (14.4)	22.5 (12.0)	<0.001
DASH Score (Diet quality: range 0–9)	3.87 (1.2)	3.97 (1.2)	0.11	4.0 (1.3)	4.1 (1.2)	0.10
Current Smoker	31 (3.9%)	31 (4.6%)	0.52	179 (4.1%)	118 (3%)	0.25
Physical Activity Level			0.46			0.84
• Sedentary	36 (6.8%)	22 (4.8%)		328 (9.6%)	305 (9.8%)	
• Less Active	176 (33.1%)	149 (32.7%)		1252 (36.7%)	1175 (37.9%)	
• Moderately Active	180 (33.8%)	150 (32.9%)		1003 (29.4%)	864 (27.9%)	
• Highly Active	140 (26.3%)	135 (29.6%)		829 (24.3%)	757 (24.4%)	
Diabetes Status			<0.001			<0.001
• Normal glucose tolerance	481 (63.5%)	376 (58.4%)		1913 (46.9%)	1590 (42.6%)	
• Prediabetes	237 (31.3%)	189 (29.3%)		1802 (44.2%)	1453 (39%)	
• Diabetes	40 (5.3%)	79 (12.3%)		364 (8.9%)	686 (18.4%)	
Calendar year of baseline visit	1990 (1984–2008)	1990 (1984–2008)		1990 (1984–2008)	1990 (1984–2008)	
Years from baseline to final visit	8.8 (0, 27.4)	10.4 (0, 27.5)		8.8 (0, 27.4)	10.4 (0, 27.5)	
Number of Visits	5 (1, 19)	5 (1, 20)		5 (1, 19)	5 (1, 20)	
Number of person-visits for each outcome:						
• Body Mass Index				4324	3944	
• Waist Circumference				4015	3711	

Data presented as mean (SD), number (%), or median (min, max).

During the study period (1984–2012), the 1454 participants contributed 3096 food diary records with 92 unique low-calorie sweetener use histories which were classified into 19 patterns defined by length and number and type of transition (S1 Table). Fifty-three percent of participants contributed a food diary at more than one visit. Of the 785 participants classified as low-calorie sweetener non-users at baseline, 628 (80%) did not report low-calorie sweetener use at any contributed food diary; of the 669 participants classified as low-calorie sweetener users at baseline, 521 (78%) reported low-calorie sweetener use at all contributed food diaries; and the remaining 305 participants (21%) switched between using and not using low-calorie sweetener during the observation period with 72 (5%) switching more than twice between statuses. A heat-map illustrates the patterns of low-calorie sweetener consumption over time (S1 Fig).

### Low-Calorie Sweetener Use and Body Size Changes

Participants who always reported using low-calorie sweetener had higher average BMI and WC (Table 2), and a higher prevalence of abdominal obesity than participants who never reported using low-calorie sweetener (Table 3). Even when low-calorie sweetener use history was examined in relation to obesity outcomes assessed at the subsequent follow-up visit to minimize reverse causality, always users had on average 0.80 kg/m^2^ higher BMI (95% confidence interval [CI], 0.17, 1.44; *P* = 0.01) and 2.6 cm larger WC (95% CI, 0.71, 4.39; *P* = 0.007) after controlling for covariates and accounting for study drop-out and truncation by death. Similarly, compared to never users, always users had 36.7% higher prevalence of abdominal obesity (Prevalence Ratio [PR] = 1.367; 95% CI, 1.104, 1.693; *P* = 0.004). The prevalence of obesity did not significantly differ according to low-calorie sweetener use history. These findings are consistent with those based on baseline low-calorie sweetener use.

**Table 2:** Relation between low-calorie sweetener consumption (baseline use and time-varying use) and measures of body size.

	Baseline low-calorie sweetener use				Time-varying low-calorie sweetener use			
Outcome	Model	Mean Difference^a^	95% CI	*P*-value	Model	Mean Difference^b^	95% CI	*P*-value
**BMI**	Model 1	0.713	(0.125, 1.301)	0.02	Model 2	0.803	(0.167, 1.440)	0.01
**Waist Circumference**	Model 1	2.553	(0.843, 4.264)	0.003	Model 2	2.547	(0.707, 4.387)	0.007

Model 1: Marginal structural model regressing outcome at **visits *after* baseline** on baseline low-calorie sweetener use controlling for baseline covariates including baseline values of the outcomes.

Model 2: Marginal structural model regressing outcome at visit v on proportion of low-calorie sweetener use up to visit v-1 controlling for covariates including the value of the outcomes at visit v-1.

^a^ Mean difference comparing low-calorie sweetener users to low-calorie sweetener non-users.

^b^ Mean difference per 100% difference in low-calorie sweetener use (i.e., always users versus never users). To compare participants whose low-calorie sweetener use differed by p%, compute Mean Difference × p/100. For example to compare mean BMI for participants whose low-calorie sweetener use differed by 25% using Model 2, compute 0.913 × 25/100 = 0.228 mean difference.

All models additionally adjusted for year of visit, age, sex, age by sex interaction, race, current smoking status, dietary intake (caffeine, fructose, protein, carbohydrate, and fat), physical activity, diabetes status (no, yes, pre-diabetes) and Dietary Approaches to Stop Hypertension (DASH) score as confounders.

**Table 3:** Relation between low-calorie sweetener consumption (baseline use and time-varying use) and measures of obesity and abdominal obesity.

	Baseline low-calorie sweetener use				Time-varying low-calorie sweetener use			
Outcome	Model	Prevalence Ratio^a^	95% CI	*P*-value	Model	Prevalence Ratio^b^	95% CI	*P*-value
**Obesity** (BMI ≥30 kg/m^2^)	Model 1	1.297	(0.982, 1.714)	0.07	Model 2	1.290	(0.939, 1.773)	0.11
**Abdominal Obesity** (WC>102cm men; WC>88cm women)	Model 1	1.448	(1.193,1.757)	<0.001	Model 2	1.367	(1.104, 1.693)	0.004

Model 1: Marginal structural model regressing outcome at **visits *after* baseline** on baseline low-calorie sweetener use controlling for baseline covariates including baseline values of the outcomes.

Model 2: Marginal structural model regressing outcome at visit v on proportion of low-calorie sweetener use up to visit v-1 controlling for covariates including the value of the outcomes at visit v-1.

^a^ Prevalence ratio per comparing low-calorie sweetener users to low-calorie sweetener non-users.

^b^ Prevalence ratio per 100% difference in low-calorie sweetener use (i.e., always users versus never users). To compare participants whose low-calorie sweetener use differed by p%, compute Prevalence Ratio^(p/100)^. For example to compare the prevalence of obesity for participants whose low-calorie sweetener use differed by 25% using Model 2, compute 1.315^(25/100)^ = 1.071 prevalence ratio.

All models additionally adjusted for year of visit, age, sex, age by sex interaction, race, current smoking status, dietary intake (caffeine, fructose, protein, carbohydrate, and fat), physical activity, diabetes status (no, yes, pre-diabetes) and Dietary Approaches to Stop Hypertension (DASH) score as confounders.

**Fig 2:**
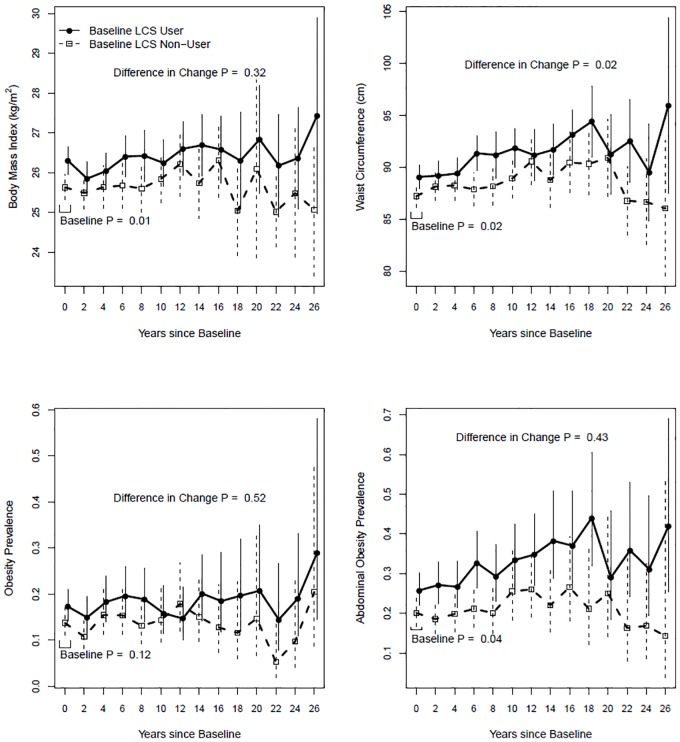
Adjusted mean differences in body size over time by baseline low-calorie sweetener use. The top panels show body mass index and waist circumference of low-calorie sweetener (LCS) user (filled circle) and non-user (open square) over time. The bottom panels show the prevalence of obesity and abdominal obesity of LCS user and non-user over time. The analysis was adjusted for the covariates mentioned in the Methods section.

In a sensitivity analysis that included time-by-baseline low-calorie sweetener use interactions, low-calorie sweetener users had higher BMI and WC at baseline; and greater adjusted increases over time in WC but not BMI, compared to non-users (Fig 2). The prevalence of abdominal obesity was higher at baseline among users than non-users and the difference did not change over time whereas the prevalence of obesity between the two groups did not differ at baseline or change over time (Fig 2).

### Low-Calorie Sweetener Use and Onset of Obesity and Abdominal Obesity

**Fig 3:**
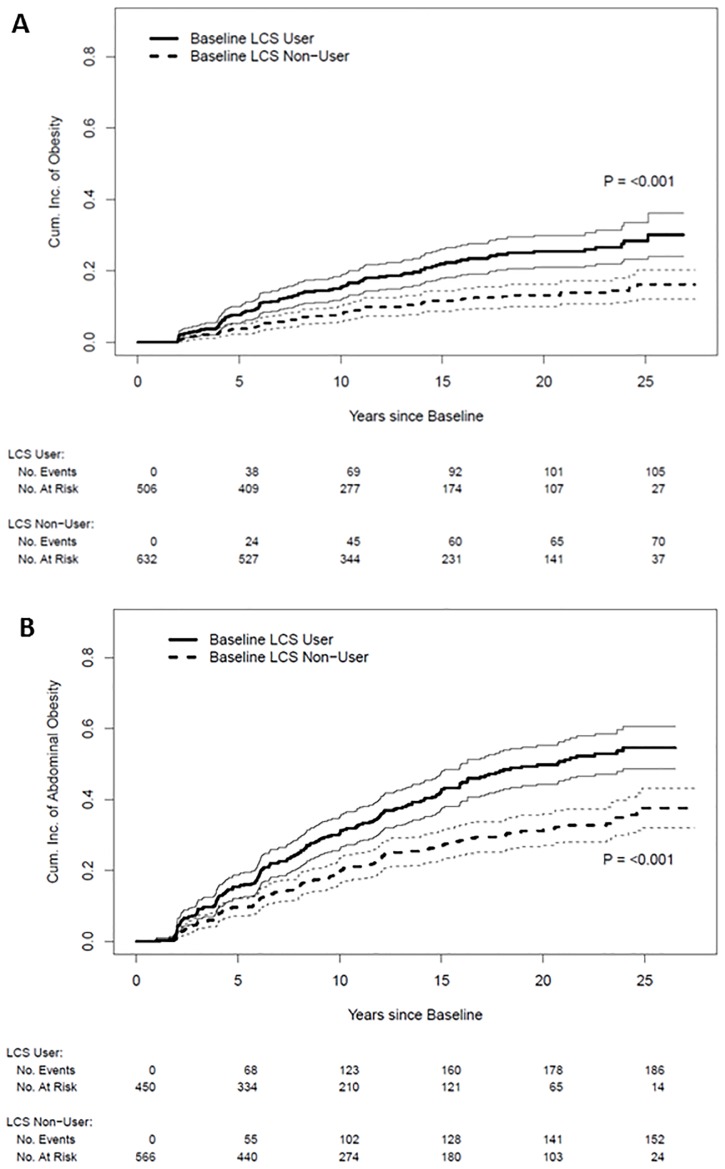
Cumulative incidence of obesity and abdominal obesity. (A) Cumulative incidence of obesity (BMI≥30mg/kg^2^); (B) cumulative incidence of abdominal obesity (WC>102cm men and WC>88cm women). Bold lines are the cumulative incidence; un-bold lines are 95% confidence intervals.

Participants who reported low-calorie sweetener use and did not have obesity at baseline had a significantly greater cumulative incidence of obesity (*P*<0.001) than participants who did not report baseline low-calorie sweetener use (Fig 3A). Similarly, compared to low-calorie sweetener non-users, users with no abdominal obesity at baseline also had a significantly higher cumulative incidence of abdominal obesity (Fig 2B). These findings were essentially confirmed after adjustment for covariates and accounting for study drop-out and truncation by death (Table 4). Assessing time-varying low-calorie sweetener use, always users had a marginally higher adjusted hazard of obesity (Hazard Ratio [HR] = 1.60; 95% CI 0.96, 2.64; *P* = 0.07) and a significantly higher adjusted hazard of abdominal obesity (HR = 1.53; 95% CI 1.10, 2.12; *P* = 0.012) than never users (Table 4).

**Table 4:** Relation of baseline and time-varying low-calorie sweetener use with onset of obesity and abdominal obesity.

Outcome	Low-calorie sweetener use	Hazard Ratio	95% CI	P-value
Obesity (BMI ≥30 kg/m^2^)	Baseline^a^	1.52	(1.11, 2.07)	**0.009**
	Proportion over time^b^	1.60	(0.96, 2.67)	0.07
Abdominal Obesity (WC>102cm men; WC>88 cm women)	Baseline^a^	1.47	(1.18, 1.83)	**0.001**
	Proportion over time^b^	1.53	(1.10, 2.12)	**0.012**

^a^ Marginal structural Cox proportional hazards model adjusted for baseline covariates. Hazard ratio compares participants who reported low-calorie sweetener use at baseline to participants who did not report low-calorie sweetener use at baseline

^b^ Marginal structural Cox proportional hazards model of time—to-outcome *after* visit v regressed on proportion of low-calorie sweetener use up to and including visit v controlling for time-varying covariates including body size measures at visit v. Hazard ratio is for a 100% difference in low-calorie sweetener use. To compare participants whose low-calorie sweetener use differed by p%, compute Hazard Ratio^(p/100)^. For example, to compare the hazard of obesity for participants whose low-calorie sweetener use differed by 25%, compute 1.64^(25/100)^ = 1.13 hazard ratio.

## Discussion

In a community volunteer cohort study of 1454 men and women with 8268 person-visits over 28 years, low-calorie sweetener use was associated with heavier weight, a larger waist, and a higher prevalence and incidence of abdominal obesity—even after accounting for diet quality and weight and body size at the time of low-calorie sweetener use assessment, which may drive the decision to consume low-calorie sweetener. These data suggest that low-calorie sweetener consumption may deleteriously affect visceral fat deposition, a strong risk factor for cardiovascular disease and mortality.

Although the hypothesis that consuming low-calorie sweeteners contributes to increased weight and fatness has been previously investigated [19, 21, 23, 27–29], the current study takes advantage of the extensive data collected in the BLSA with up to 28 years of longitudinal observations on body composition and low-calorie sweetener consumption found in all food products, not just diet soda. Further, two different measures of adiposity—obesity by BMI and abdominal obesity by WC—all showed chronic low-calorie sweeteners consumption was associated with increasing obesity, most noticeably abdominal obesity. These findings have important public health implication since the prevalence of abdominal obesity in the US continues to rise. Our finding of low-calorie sweetener use and weight gain is consistent with results from two longitudinal studies, with six to nine years of follow-up, reporting an association between consumption of low-calorie sweetener containing beverages and increased weight gain [19, 23]. The finding of increase in waist circumference with low-calorie sweetener use is consistent with prior work on the association between diet soda with increased waist circumference [21, 23]. In our study, the extensive longitudinal information allowed us to account for time-varying low-calorie sweetener use and thereby strengthen the evidence of a potential causal relationship.

Recent longitudinal studies suggest that risk of increased waist circumference and other cardiometabolic outcomes associated with low-calorie sweeteners may be modified by dietary quality [27–29]. Specifically, non-users of diet soda have smaller waistline than users regardless of diet quality and furthermore, individuals who consume a “western” diet with diet soda have significantly larger waistline compared to individuals on “prudent” diet with no diet soda [29]. In our analyses, taking dietary quality into account further strengthen the association between low-calorie sweetener use and risk of gaining weight, increasing waistline, and incidence and prevalence of abdominal obesity (results not shown). This finding suggests that diet quality may be a negative confounder in the association between low-calorie sweetener use and obesity.

Over the years, controlled intervention trials of 10 weeks to 12 months duration, have reported that low-calorie sweetener beverages results in modest weight loss or has a negligible effect [10–17]. Substituting low-calorie beverages for sugar-sweetened beverages can decrease energy intake, but evidence of their effectiveness for weight management is limited. Low-calorie sweeteners certainly have fewer calories; however, our study suggests that they may have metabolic activity that is pro-obesogenic—especially for abdominal adiposity—and this hypothesis should be actively researched.

Mechanisms for the association between low-calorie sweetener use and progressively rising prevalence of abdominal obesity remain unknown. One potential explanation derives from the physiology of the brain food reward system. Evidence from animal studies demonstrates that dopamine signaling in the brain may function as a “central caloric sensor” that regulates nutrient intake accounting for the caloric density of food [40]. Low-calorie sweeteners used as a sugar alternative may reduce food specific calories, but because they do not produce satiety, they can encourage more eating. Ageusic mice, lacking sweet taste sensation due to mutation in the taste ion channel TRPM5, acquire preference for sucrose and show robust dopamine release with sucrose but not with low-calorie sweetener ingestion [41]. This finding exemplifies the hypothesis that the reward system in the brain normally links “sweetness” with absorption of calories in the gut. Low-calorie sweeteners, with no caloric density, actually may cause the brain to abandon sweetness as a calorie gauge. Therefore, individuals who consume low-calorie sweeteners may compensate by over-eating in order to experience the expected satiety. Another possible mechanism may involve the gut microbiome. Evidence from a rodent study indicates that chronic saccharin consumption contributes to weight gain and worsening glucose tolerance by inducing dysbiosis and altering the composition and function of intestinal microbiota. The altered microbiota exhibit enhanced energy harvest pathways previously associated with obesity in mice and humans. These mice also had altered bacterial taxa similar to those found associated with type 2 diabetes in humans [42, 43]. Other low-calorie sweeteners such as sucralose has also been shown to contribute to weight gain and altered intestinal microbiota in rats [44]. Low-dose aspartame, however, was associated with altered intestinal microbiota with worsening glucose tolerance but less weight gain in rats when challenged with high fat diet [45]. The underlying mechanism is unknown and warrants further investigation.

The strengths of our study include the follow-up period of up to 28 years and the use of 7-day food diaries at multiple follow-up visits. The food diary provides details of low-calorie sweetener use in all food products including coffee, tea, and dessert, which allow for a more comprehensive assessment of low-calorie sweetener consumption beyond diet soda. Previous studies assessed diet soda consumption using food frequency questionnaires with participant assessment done at study centers [19–24] or using mailed questionnaires [25, 26]. In addition, we took into consideration diet quality using the DASH score. Lastly, since we accounted for body composition at the time of assessment of low-calorie sweetener use, the possibility that risk of weight gain largely explains the observed associations between low-calorie sweetener use and forms of obesity is minimized.

Using different models and approaches to account for initial “indication” and changing usage patterns, we consistently found low-calorie sweetener use associated with weight gain and expanding waistline. Nevertheless, several limitations should be considered in interpreting the findings. First, BLSA is an observation study, therefore we cannot rule out the possibility of unmeasured confounders. However, we mitigated this possibility by including many relevant covariates and by analytically accounting for other factors. For example, we modeled visits after low-calorie sweetener assessment to minimize reverse causality and modeled the drop-out and mortality process to reduce bias from missing data. We also took into account dietary quality as a potential confounder. Second, the BLSA cohort is, in general, a highly motivated and health conscious group of individuals; for example, only 17% of the cohort developed obesity over the follow-up period, compared to the US adult population with obesity prevalence of 38% in 2014 [2]. However, this limitation can also be considered a study strength in that weight gain and increasing waistline were demonstrated with low-calorie sweetener consumption even in this highly motivated cohort. Third, low-calorie sweetener use is represented as a dichotomous variable (yes or no), thus we do not have a more granular measure of low-calorie intake and our estimates are likely conservative. Finally, the BLSA cohort are older with baseline age of about 60 years; therefore, the results may be subject to survival bias and may not be generalizable to younger adults. To mitigate the degree of bias, we used inverse probability of survival weights in our models to account for factors that may jointly impact study outcomes and mortality.

Our finding has important public health implications. These findings underscore that weight management strategies should be rooted in understanding how the human body responds to certain types of food instead of merely considering the theoretical caloric content. Independent of caloric content, the specific food eaten affects subsequent eating behavior, influences intestinal microbiota that contributes to energy handling and interacts with enteroendocrine and neuroendocrine pathways, all mechanisms which ultimately affect energy homeostasis. Through these mechanisms, low-calorie sweetener use may be a contributor to the obesity epidemic.

## Supporting Information

S1 Fig Heat map illustrating patterns of low-calorie sweetener consumption in participants over time. n = 1,454 participants from 1984 to 2012. Orange = self-reported non-LCS use; Blue = self-report LCS use; White = visit with no diary record; Gray = no visit.
(DOCX)

S1 Table Low-calorie sweetener use history patterns and characteristics by pattern. (DOCX)
