# Supplementary material for: Chronic Low-Calorie Sweetener Use and Risk of Abdominal Obesity among Older Adults: A Cohort Study
Source: PLoS One. 2016 Nov 23;11(11):e0167241. doi: 10.1371/journal.pone.0167241 (PMC5120853; doi:10.1371/journal.pone.0167241)
Supplement: S1 Fig — n = 1,454 participants from 1984 to 2012. Orange = self-reported non-LCS use; Blue = self-report LCS use; White = visit with no diary record; Gray = no visit. (DOCX) [file pone.0167241.s001.docx]

**S1 Fig. Heat map illustrating patterns of low-calorie sweetener consumption in participants over time.** n = 1,454 participants from 1984 to 2012. Orange = self-reported non-LCS use; Blue = self-report LCS use; White = visit with no diary record; Gray = no visit.

**
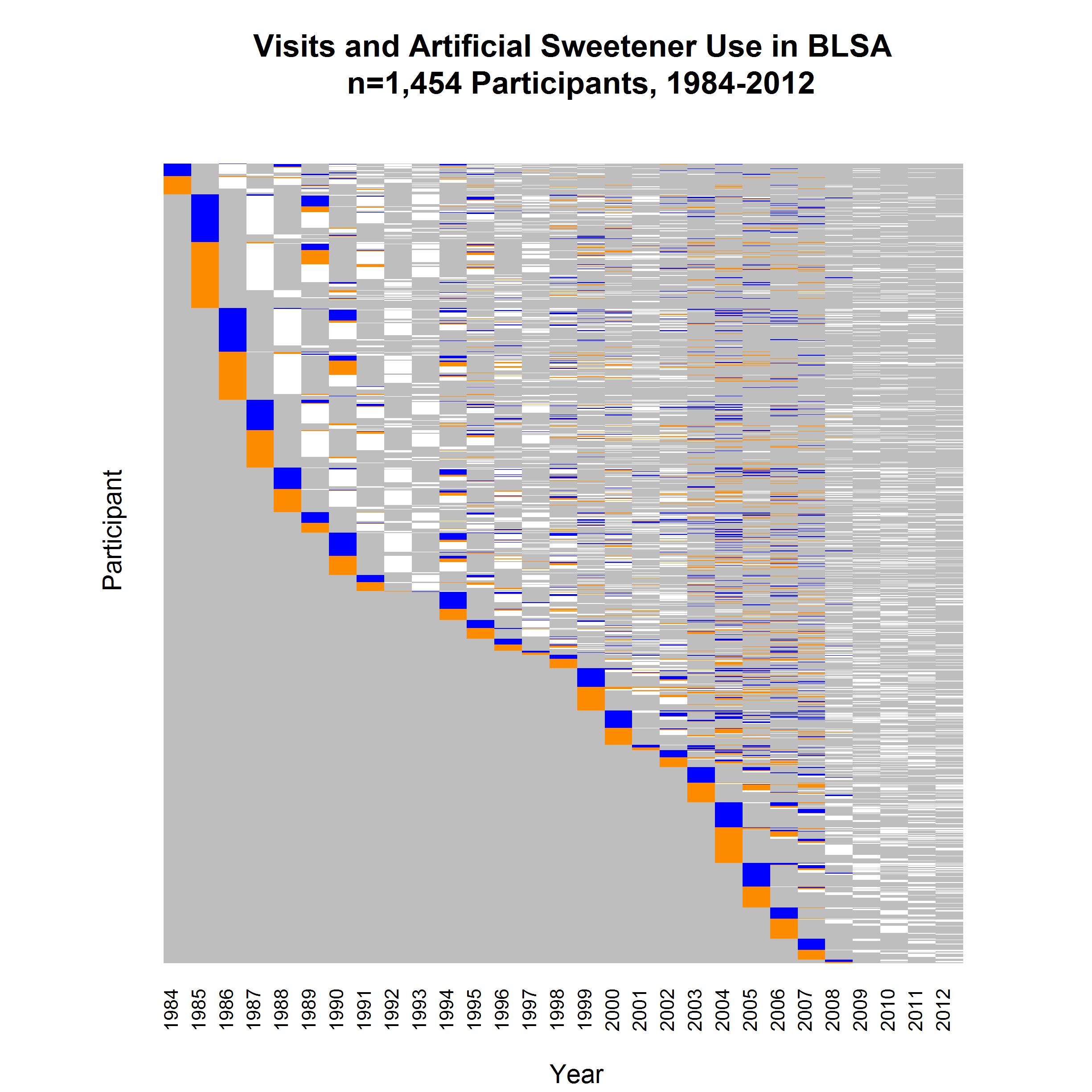
**
