# Supplementary material for: Chronic Low-Calorie Sweetener Use and Risk of Abdominal Obesity among Older Adults: A Cohort Study
Source: PLoS One. 2016 Nov 23;11(11):e0167241. doi: 10.1371/journal.pone.0167241 (PMC5120853; doi:10.1371/journal.pone.0167241)
Supplement: S1 Table — (DOCX) [file pone.0167241.s002.docx]

**S1 Table. Low-calorie sweetener use history patterns and characteristics by pattern**

| **Low-calorie sweetener pattern length** | **Low-calorie Sweetener Pattern^a^** | **N** | **Year of Baseline Visit,**  **Median (Min, Max)** | **Baseline Age (yr),**  **Mean (SD)** | **Baseline BMI (kg/m^2^),**  **Mean (SD)** | **Male,**  **N (%)** | **No.**  **Person-Visits** |
| --- | --- | --- | --- | --- | --- | --- | --- |
| 1 | 0 | 382 | 1996 (1984, 2008) | 61.6 (17) | 25.6 (4.3) | 197 (51.6%) | 1254 |
|  | 1 | 304 | 1995 (1984, 2008) | 60 (17) | 26.9 (4.8) | 151 (49.7%) | 1038 |
| 2 | 00 | 134 | 1991 (1984, 2006) | 62.1 (16.4) | 24.9 (3.3) | 74 (55.2%) | 727 |
|  | 01 | 47 | 1991 (1984, 2005) | 63.6 (15.6) | 25.3 (3.5) | 16 (34%) | 264 |
|  | 10 | 44 | 1995 (1985, 2006) | 59.6 (17.9) | 27.3 (5.6) | 24 (54.5%) | 242 |
|  | 11 | 115 | 1994 (1984, 2005) | 58.6 (16.3) | 26.3 (4.5) | 55 (47.8%) | 666 |
| 3 | 000 | 63 | 1994 (1984, 2004) | 64.7 (12.2) | 25.2 (3.9) | 27 (42.9%) | 469 |
|  | 001 | 18 | 1989 (1985, 2004) | 61.9 (17.9) | 26 (4.2) | 8 (44.4%) | 146 |
|  | 010 | 12 | 1990 (1985, 2003) | 61.2 (11.4) | 23.8 (3.2) | 6 (50%) | 91 |
|  | 100 | 17 | 1989 (1984, 1999) | 60.2 (14.3) | 24.1 (2.6) | 10 (58.8%) | 139 |
|  | 011 | 16 | 1986 (1985, 2002) | 60.2 (14.2) | 25.4 (3.8) | 10 (62.5%) | 115 |
|  | 101 | 9 | 1986 (1984, 2002) | 50.8 (14.1) | 24.6 (4.2) | 4 (44.4%) | 75 |
|  | 110 | 14 | 1988 (1986, 2004) | 59.8 (15.7) | 26.4 (4.4) | 10 (71.4%) | 116 |
|  | 111 | 42 | 1993 (1984, 2005) | 60.7 (12.2) | 26.1 (3.8) | 20 (47.6%) | 332 |
| >=4 | 0...0 | 49 | 1987 (1984, 2003) | 61.6 (13.9) | 24.8 (4) | 27 (55.1%) | 506 |
|  | 1...1 | 60 | 1988 (1984, 2002) | 57.5 (11.8) | 25.6 (3.8) | 31 (51.7%) | 610 |
|  | 0...01...1 | 25 | 1987 (1985, 1999) | 57.1 (12.3) | 25.1 (4.2) | 17 (68%) | 275 |
|  | 1...10...0 | 31 | 1988 (1984, 1999) | 62.6 (13.8) | 26.1 (3.3) | 18 (58.1%) | 336 |
|  | >= 2 transitions | 72 | 1987 (1984, 2001) | 59.5 (11.5) | 25.2 (3.5) | 36 (50%) | 872 |

^a^0 refers to low-calorie sweetener non-user; 1 refers to low-calorie sweetener user. 0…0 refers to low-calorie sweetener non-use over an arbitrary number of visits; 1…1 refers to low-calorie sweetener use over an arbitrary number of visits.
